# Effects of Caffeine Consumption on Attention Deficit Hyperactivity Disorder (ADHD) Treatment: A Systematic Review of Animal Studies

**DOI:** 10.3390/nu14040739

**Published:** 2022-02-10

**Authors:** Javier C. Vázquez, Ona Martin de la Torre, Júdit López Palomé, Diego Redolar-Ripoll

**Affiliations:** 1 Faculty of Psychology and Educational Sciences, Cognitive NeuroLab, Universitat Oberta de Catalunya, 08018 Barcelona, Spain; odominguezma@uoc.edu (O.M.d.l.T.); dredolar@uoc.edu (D.R.-R.); 2 Neuromodulation Unit, Institut Brain 360, 08022 Barcelona, Spain; 3 Consorci d’Educació de Barcelona, Centre de Màxima Complexitat Elisenda de Montcada, Generalitat de Catalunya, 08010 Barcelona, Spain; jlope284@xtec.cat

**Keywords:** caffeine, attention deficit hyperactivity disorder, impulsivity, ADHD, animal models

## Abstract

Attention deficit hyperactivity disorder (ADHD) is a neurodevelopmental disorder characterized by a persistent pattern of inattention and/or hyperactivity-impulsivity. ADHD impairments arise from irregularities primarily in dopamine (DA) and norepinephrine (NE) circuits within the prefrontal cortex. Due to ADHD medication’s controversial side effects and high rates of diagnosis, alternative/complementary pharmacological therapeutic approaches for ADHD are needed. Although the number of publications that study the potential effects of caffeine consumption on ADHD treatment have been accumulating over the last years, and caffeine has recently been used in ADHD research in the context of animal models, an updated evidence-based systematic review on the effects of caffeine on ADHD-like symptoms in animal studies is lacking. To provide insight and value at the preclinical level, a systematic review based on PRISMA guidelines was performed for all publications available up to 1 September 2021. Caffeine treatment increases attention and improves learning, memory, and olfactory discrimination without altering blood pressure and body weight. These results are supported at the neuronal/molecular level. Nonetheless, the role of caffeine in modulating ADHD-like symptoms of hyperactivity and impulsivity is contradictory, raising discrepancies that require further clarification. Our results strengthen the hypothesis that the cognitive effects of caffeine found in animal models could be translated to human ADHD, particularly during adolescence.

## 1. Introduction

Attention Deficit Hyperactivity Disorder (ADHD) is the most commonly diagnosed and treated mental disorder during childhood [1] and it is increasingly diagnosed and treated in during adulthood [2]. ADHD is a neurodevelopmental disorder characterized by a pattern of inattention and/or hyperactivity-impulsivity, persisting no less than six months, that is inconsistent with developmental level and has negative impact in at least two settings (academic, occupational or social) [3]. Inattention refers to important difficulties in sustaining attention to tasks that do not deliver a high level of stimulation or regular rewards, distractibility, and difficulties with organisation. Hyperactivity refers to disproportionate motor activity and difficulties with remaining still, most manifest in structured situations that involve behavioral self-control. Finally, impulsivity is a propensity to behave in response to immediate stimuli, without consideration of the risks and consequences [4]. Specific manifestations vary across individuals, and may change over the course of development. Depending on the symptoms presented, three different types of ADHD can be diagnosed: predominantly inattentive presentation, predominantly hyperactive-impulsive presentation, or combined presentation [3,4]. Although ADHD onset occurs during childhood and it often persists into adulthood, there is an important knowledge gap concerning ADHD lifespan aspects [5]. Population surveys suggest that ADHD occurs in most cultures in about 5% of children and about 2.5% of adults [6] and, as of 2019, it was estimated to affect 84.7 million people worldwide [7]. ADHD management recommendations depend on the country [8,9,10] and usually include psychotherapy (essentially Cognitive Behavior Therapy, CBT), lifestyle changes and medications [11]. ADHD medication treatment, however, has been historically considered controversial [12], particularly due to its side effects [13,14,15]. In the face of these controversies and high rates of diagnosis, alternative/complementary pharmacological therapeutic approaches for ADHD are needed.

Although larger ADHD models containing supplementary pathways have been suggested [16,17], it is widely accepted that ADHD impairments, including selective and sustained attention, impulsivity, and motor activity, arise from abnormalities in different circuits involving the prefrontal cortex [18]: sustained attention is modulated by a cortico-striato-thalamocortical (CSTC) loop that comprises the dorsolateral prefrontal cortex (DLPFC) projecting to the striatal complex. Selective attention is modulated by a cortico-striato-thalamo-cortical (CSTC) loop ascending from the dorsal anterior cingulate cortex (dACC) and projecting to the striatal complex, followed by the thalamus, and back to the dACC. Impulsivity is related to a cortico-striato-thalamocortical (CSTC) loop that contains the orbitofrontal cortex (OFC), the striatal complex, and the thalamus. Finally, motor activity, including hyperactivity and psychomotor agitation or retardation, can be modulated by a cortico-striato-thalamo-cortical (CSTC) loop arising from the prefrontal motor cortex to the lateral striatum to the thalamus and back to the prefrontal motor cortex. ADHD patients cannot activate prefrontal cortex areas in an appropriate manner when responding to cognitive tasks requiring attention and executive control, and show a dysfunction in reward and motivation, hindering cognitive control of behaviour [19,20]. Children diagnosed with ADHD, in this regard, need stronger incentives to adapt their behaviour [21], showing impaired responses to partial schedules of reinforcement and difficulties in delaying gratification [22,23].

In ADHD, inefficient information processing and arousal-related behaviours are hypothetically caused by imbalances mainly in the dopamine (DA) and norepinephrine (NE) circuits [24,25] and the serotonin (5-HT), glutamate (GLU), and acetylcholine (ACh) pathways within these areas of the brain [26,27,28].

Different genes are associated with the disorder, including the serotonin transporter (SERT), the synaptosomal-associated protein (SNAP-25), and the brain-derived neurotrophic factor (BDNF) [29,30], while some genes directly affect DA neurotransmission, including the DA transporter (DAT) or the DA receptor 4 (DRD4) [31,32]. In this respect, the ventral tegmental area (VTA) and locus coeruleus (LC) neurons have different targets, although their efferent fibers converge into the PFC: DA is released into the nucleus accumbens (NAcc), facilitating reward; NE is released in different posterior cortical areas, optimizing the organism reaction to significant stimuli; and both organic compounds are released into the PFC, enhancing working memory and attention in the face of significant stimuli [33].

**Figure 1:**
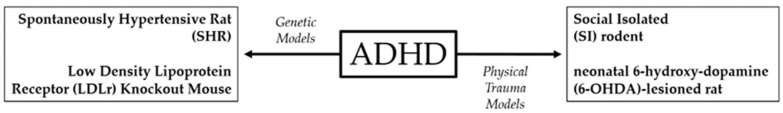
Animal models of Attention Deficit Hyperactivity Disorder. Key for abbreviations used: SHR: spontaneously hypertensive rat, low-density lipoprotein receptor, SI: social isolated, 6-OHDA: 6-hydroxy-dopamine, ADHD: Attention Deficit Hyperactivity Disorder.

Animal studies have provided insights into the pathological and neurochemical basis of ADHD through different types of animal model (see Figure 1) [34]. Among these, the spontaneously hypertensive rat (SHR) is considered an excellent and validated hyperactive model to study ADHD. Concerning its behavioral profile, SHR presents anomalies in DA neurotransmission [35] and, importantly, in adenosine neurotransmission [36].

Caffeine, in this respect, is an adenosine A_1_ and A_2A_ receptor antagonist controlling synaptic plasticity [37]. These receptors are functionally paired with certain postsynaptic DA receptors, such as D2 receptors, where DA binds and has a stimulatory effect. When adenosine binds to its receptors, this causes reduced sensitivity of D2 receptors. Antagonism of adenosine receptors by caffeine prevents adenosine from binding, enhancing dopaminergic actions [18,24]. In addition to these dopaminergic effects, it has been shown that caffeine also produces secondary effects on ACh and NE [37,38,39]. Moreover, caffeine’s effects on the non-selective antagonism of adenosine receptors also generate vasoconstriction in the nervous system. In this respect, it has been shown that caffeine modifies the blood perfusion signal, measured by fMRI, due to its neural and vascular effects, depending on the cerebral distribution of its receptors [40]. Similarly, the effect that caffeine may have at the cognitive level could depend on its regional effects on vascular response [41].

Nevertheless, the potential of caffeine consumption as a treatment for ADHD remains largely controversial, with studies showing efficacy in relieving ADHD-related symptoms [42], and studies failing to find superior effects when compared to first-line ADHD medication [43]. Beyond ADHD, there is an existing correlation between the daily consumption of moderate doses of caffeine and related benefits in different psychiatric disorders linked with adenosine A_2A_ receptor blockade controlling synaptic plasticity [44], mainly at the glutamatergic synapses [45]. Moreover, regular coffee consumption improves children’s performance in comparison to decaffeinated coffee or placebo [46]. However, some studies have reported that caffeine consumption improvement is not significantly superior to placebo [47] or methylphenidate (MPD) [48], while hyperactivity has been strongly associated with higher coffee consumption among adolescents [49].

**Figure 2:**
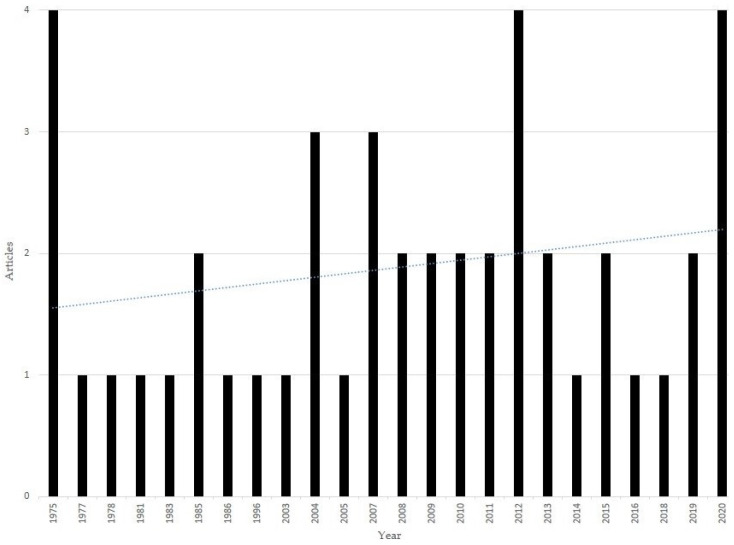
Caffeine/Attention Deficit Hyperactivity Disorder-related articles since 1975 (Source: MEDLINE).

The number of publications that study the potential effects of caffeine consumption on ADHD treatment has accumulated since 1975 (see Figure 2) and, over the last few years, caffeine has been used in ADHD research in the context of animal models. Surprisingly, an updated evidence-based systematic review on the effects of caffeine on ADHD-like symptoms in animal studies is lacking.

Consequently, to provide insight and value at the preclinical level, we sought to produce a comprehensive compilation and systematically review all the relevant scientific publications that make reference to the underlying effects of caffeine intake on treating ADHD-like symptoms in animal studies.

## 2. Materials and Methods

We conducted a systematic review of ADHD research in the context of animal models to assess the association between caffeine and ADHD-dependent variables including attention, locomotor activity, impulsive behavior, learning, and memory.

### 2.1. Search Strategy

**Figure 3:**
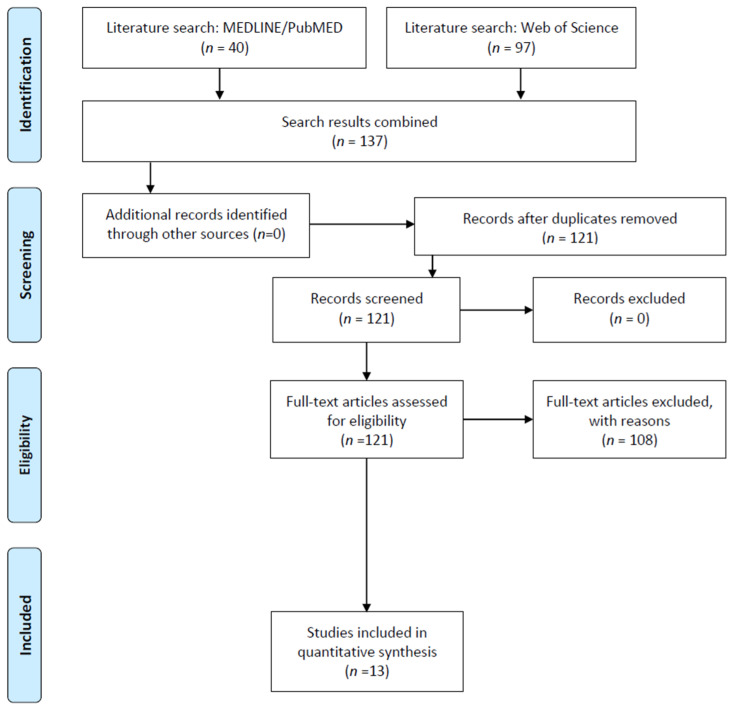
Flow diagram of study’s selection based on PRISMA guidelines [50].

Figure 3 depicts the search strategy. We followed the guidelines and recommendations contained in the Preferred Reporting Items for Systematic Reviews and Meta-Analyses (PRISMA) statement [50], in order to reliably structure the gathered information in this systematic review. Academic articles were located using two electronic databases: MEDLINE and Web of Science. Only the results from these two databases were reported, since results from other sources (Scopus, Google Scholar) did not provide any relevant new results. No restrictions regarding publication date were applied. The literature search was conducted on 5 September 2021.

According to our proposal, the MEDLINE search strategy was established on the following key search terms: “caffeine” [Mesh] AND “Attention Deficit Disorder with Hyperactivity” [Mesh]. MeSH (Medical Subject Headings) terms were therefore used in the development of this search. The Web of Science search strategy was based on the following key search terms: (“attention” OR “hyperactivity” OR “ADHD”) AND “caffeine”.

### 2.2. Study Selection Criteria

The search was limited to preclinical and original experiments on non-human animals. The inclusion criteria were: (1) English-written, indexed studies; (2) non-human animal preclinical/experimental studies; (3) the mention of the relationship between caffeine treatment and ADHD-like symptoms; and (4) controlled studies with separately treated groups. The exclusion criteria were: (1) clinical/experimental/qualitative studies on humans; (2) not mentioning caffeine treatment and ADHD-like symptoms at all; (3) reviews, posters, conference abstracts, oral speeches, commentaries, theoretical papers, unpublished relevant studies, and other studies relevant to the topic but not published in peer-reviewed journals; and (4) case and cross-over studies.

### 2.3. Study Selection

Duplicates of all the databases were removed. Titles and abstracts were independently screened by two authors (J.C.V. and O.M.d.l.T.) according to the inclusion and exclusion criteria. Articles interpreted as compatible were selected for a full-text analysis to determine whether they were or were not within the inclusion criteria. Furthermore, the references of selected studies were screened in search of additional articles that met the inclusion criteria. Whenever a divergence of opinions emerged, a third author (D.R.-R.) was consulted to discuss and reach an agreement between the authors.

### 2.4. Data Extraction and Analyses

Following selection of the studies, data were extracted and prearranged into a table. The subsequent information was collected: (1) author/s and year; (2) species: strain, sex, and sample (*n*); (3) animal model; (4) age; (5) independent variables; (6) caffeine treatment; (7) behavioral tests/types of stress; (8) dependent variables; and (9) main results.

## 3. Results

Due to the large number of results acquired by the search terms, strict inclusion/exclusion criteria were applied to limit the final selection of studies. Figure 2 shows the studies included in quantitative synthesis.

### 3.1. Study Selection

A total of 121 unique citations was initially retrieved through the combined search, after which 108 citations were excluded after full-text screening because they did not meet the inclusion criteria. Therefore, 13 studies (Pandolfo et al., 2013 [51]; Ouichi et al., 2013 [52]; Caballero et al., 2011 [53]; Ruiz-Oliveira et al., 2019 [54]; Higgins et al., 2007 [55]; França et al., 2020 [56]; Nunes et al., 2018 [57]; Szczepanik et al., 2016 [58]; Pires et al., 2010 [59]; Prediger et al., 2005 [60]; Leffa et al., 2019 [61]; Pires et al., 2009 [62]; Alves et al., 2020 [63]) on animal models were finally considered. Based on their methodology, the studies in this review could be classified as experimental (*n* = 10; 76.9%), randomly assigning the subjects sample to the experimental groups, and quasi-experimental (*n* = 3; 23.1%), where the groups were usually constructed according to the subject’s characteristics. The first studies relevant to the topic were from 2005, while the most recent studies included in this review were published in 2020. Table 1 describes each article individually.

#### 3.1.1. Species, Animal Model, Sex, and Treatment

Most of the animal studies were performed on rodents. Ten studies were conducted with rats and two with mice. Only one of the studies used zebrafish as an animal model. Four studies used only males, five used both males and females, and four used only females. Different caffeine treatments and routes of administration were used, along with different durations (Table 1). Chronic treatments were mainly performed by dilating caffeine powder in the system water, whereas acute treatments were mainly administered intraperitoneally (i.p.).

#### 3.1.2. Animal Models of ADHD

Overall, nine studies used genetic animal models of ADHD: eight studies used SHR, and one study used the low-density lipoprotein receptor (LDLr) mouse. Finally, two studies used physical trauma to provide an epigenetic animal model of ADHD: one study caused 6-hydroxy-dopamine (6-OHDA) lesions in rats, while one study used social isolation (SI) as an intensely stressful environment in mice (Table 1).

#### 3.1.3. Behavioral Tests

Five studies used the object recognition task; four studies used the Y maze test; three studies used the open field test; two studies used the water maze test; and two studies used the novel object recognition test. In addition, other tests were performed, including the water-finding test, the five-choice serial reaction time task (5-CSRTT), the locomotor activity test, the discrimination task, the Olton maze behavioral assay, the attention set-shifting task, the fear-conditioning test, the tolerance to delay of reward task, and the olfactory discrimination test. Finally, one study induced the animals to a certain type of stress by means of social isolation and aggressivity (Table 1).

### 3.2. Study Outcomes

The results are summarized in Table 1. Considering the amount of data provided in the reviewed articles, we decided to categorize all the information based on caffeine’s effects on each relevant ADHD-like evaluated parameter, as follows.

#### 3.2.1. Attention

##### Attention and Behavioral Flexibility

Pandolfo et al. [51] examined the impact of chronic caffeine treatment during adolescence on SHR and Wistar Kyoto (WKY) rats’ performance in an attention set-shifting task, placing emphasis on response to conflict. The task was divided into different phases: familiarization, response discrimination, and visual cue discrimination. During the response discrimination phase, statistical analysis showed that vehicle-treated SHR needed a superior amount of trials to reach the benchmark of 10 consecutive correct choices, compared with WKY rats. Importantly, treatment with caffeine (2 mg/kg, i.p.) improved SHR discriminative learning in a selective manner, as indicated by a reduction in the number of trials needed to reach the benchmark, while treatment with caffeine had no effect on WKY rats. During the visual cue discrimination phase, SHR required more trials to master the task, compared with WKY rats. Once more, caffeine treatment (2 mg/kg, i.p.) diminished the number of trials needed to reach the benchmark. Finally, statistical analysis showed that vehicle-treated SHR made significantly more regressive and never-reinforced errors than vehicle-treated WKY rats. Remarkably, while treatment with caffeine (2 mg/kg, i.p.) diminished the number of these errors in SHR, it had no effect on WKY rats.

##### Spatial Attention

Ouchi et al. [52] tested the effect of SI on latent learning using the water-finding test, measuring entering latency and drinking latency. The authors eventually discussed the utility of SI as an ADHD epigenetic model. They socially isolated male or female Institute of Cancer Research (ICR) mice for one week or more. Subsequently, the animals displayed spatial attention deficit during the water-finding task. Five weeks of resocialization following one week of SI failed to improve this deficit. Drinking latency depended on how much attention the animal paid to environmental factors, including the location of a tap water nozzle, which they were exposed to in the training trial. Therefore, a decrease in drinking latency correlated with the animal remembering the position/location of the nozzle. Caffeine (0.3–1 mg/kg, i.p.) induced changes in drinking latency on the water-finding test, in this sense, significantly ameliorating SI-induced latent learning deficits in a dose-dependent manner, independently of gender or age.

Caballero et al. [53] examined caffeine’s therapeutic use in neonatal 6-OHDA lesioned rats, which constitute another existing ADHD animal model. At postnatal day (PND) 7, the rats were lesioned at the left striatum with 6-OHDA. At PND 25, spatial attention was measured with an eight arm radial maze, the Olton maze. The animals were then placed in the maze. The total number of arms the animals walked before completing six out of eight, or until they repeated one of them, was measured. After 14 days of treatment with caffeine, administered ad libitum into the drinking water, the authors assessed caffeine’s effects on the attention deficit of the animals, using the same task. Interestingly, the 6-OHDA lesioned rats significantly improved their attention deficit after caffeine treatment. Consequently, the authors highlighted the properties through which caffeine managed the attentional deficits occurring during the prepubertal period of ADHD.

##### Discrimination

Ruiz-Oliveira et al. [54] evaluated the effect of caffeine on zebrafish performance in a task requiring focus and attention, the discrimination task. The task took place in three phases: tank acclimation, training, and test. The authors used visual cues during the training trials and the test trials. Distractors, objects resembling the target, were used to confuse the fish and impair conditioning. The fish were exposed to different caffeine concentrations for 14 days: 0 mg/L (control), 10 mg/L (low), and 50 mg/L (high). Notably, low caffeine doses improved the fishes’ ability to discriminate the cues and reach the target; the fish spent most of the time close to the target where the reward was offered, and showing the shortest latency to reaching the target. The higher dose impaired the fishes’ ability to find the target; the fish demonstrated increased anxiety, a possible side effect of the substance.

##### Selective Attention

Higgins et al. [55] evaluated the effect of caffeine on Long–Evans (LE) and Cesarean-derived (CD) rat performance in a selective attention task, the 5-CSRTT. The effects of caffeine were compared to the selective A_2A_ antagonists, SCH 412348 and KW-6002, and the A_1_ antagonist, DPCPX. Caffeine (3–10 mg/kg, i.p.) increased reaction time in both LE and CD rats, with no effect on accuracy, an effect replicated by SCH 412348 (0.1–1 mg/kg PO) and KW-6002 (1–3 mg/kg PO), but not DPCPX (3–30 mg/kg PO). The faster response speed was observed in both the CD and LE rat strains at 3 mg/kg, although increased premature responses were confined to the LE strain at the 10 mg/kg dose. These results suggest that the attention-enhancing effects of caffeine were mediated through A_2A_ receptor blockade. Selective A_2A_ receptor antagonists may therefore have potential as therapies for attention-related disorders, such as ADHD.

#### 3.2.2. Hyperactivity and Impulsivity

##### Locomotor Activity

França et al. [56] tested the effect of caffeine on the hyperlocomotion characteristic of ADHD by examining locomotor activity. Caffeine consumption (0.3 mg/mL in drinking water) and physical exercise in running wheels for 6 weeks, either during adolescence (30 days old) or adulthood (4–5 months old), did not relate to changes in spontaneous locomotion in SHR, in an open field, during the 5 min habituation phase of the object recognition test. Ruiz-Oliveira et al. [54] studied the effects of caffeine on zebrafish (4 months old, wild type, both sexes). Low concentrations of caffeine (10 mg/L) affected locomotor parameters, increasing average speed and decreasing freezing behavior. Interestingly, the levels of freezing and locomotor behavior were the same for the 50 mg/L caffeine group and the control group. Nunes et al. [57] evaluated locomotor activity during the late childhood and the end of adolescence of male and female SHR, using an open field arena and measuring the total distance travelled in meters along the periphery during 5 min. Although caffeine (0.3 g/L) did not impact hyperlocomotion during late childhood (PND 28) in either sex, continuous treatment aggravated adolescent female SHR hyperactivity (PND 50), suggesting that the consumption of caffeine during childhood may aggravate hyperactivity in females, but only if the administration persists up to adolescence. Szczepanik et al. [58] demonstrated that young (3 months old) and middle-aged (8 months old) LDLr mice display different responses to chronic caffeine treatment in terms of motor activity. Although caffeine was unable to modify the hyperlocomotion observed in 3 months old LDLr mice, caffeine attenuated the increased locomotor activity observed in 8 months old LDLr mice. Pandolfo et al. [51] tested whether chronic treatment with caffeine was able to counteract the hyperlocomotion characteristic of ADHD in SH, during the open field test. Chronic treatment with caffeine did not alter central and total locomotion in SHR. Similarly, Pires et al. [59] showed that chronic treatment with caffeine did not produce changes in SHR locomotion during the object recognition task sample phase. Interestingly, Caballero et al. [53] showed that neonatal 6-OHDA lesioned rats, a different ADHD animal model, demonstrated a non-significant tendency to decrease their motor activity after ad libitum caffeine consumption throughout the prepubertal period during an Olton maze behavioral assay. Higgins et al. [55] conducted two separate types of locomotor activity study. In a CGS-21680-induced hypolocomotion assay, pretreatment with caffeine (3–30 mg/kg, i.p.) produced a significant attenuation of the CGS-21680 hypolocomotion at different doses, of 10 mg/kg and 30 mg/kg, in CD rats. In a second experiment, caffeine (1–30 mg/kg, i.p.) produced a dose-related increase in locomotion in the animals habituated to the test chambers. Finally, Prediger et al. [60] did not find a direct increase in locomotor performance in SHR after the administration of acute doses of caffeine (1–10 mg/kg i.p.) when using a spatial version of the Morris water maze. No alteration was observed in the swimming speed in this regard.

##### Impulsive Behavior

Leffa et al. [61] focused on impulsive behavior to clarify the neurobiology of ADHD. They treated SHRs with caffeine, a non-selective adenosine receptor antagonist, to assess the modulating effects of the adenosine systems on tolerance to the delay of a reward. The animals had to choose between a small, but immediate, or a large, but delayed, reward. An acute pretreatment with caffeine (2 mg/kg or 5 mg/kg) increased number of large-reward choices. Conversely, chronic treatment with caffeine (2 mg/kg, for 21 days) augmented the impulsive phenotype and decreased the number of large-reward choices.

#### 3.2.3. Learning and Memory

##### Non-Associative Learning

Habituation is a form of non-associative learning in which the animal’s innate response to a stimulus decreases after prolonged or repeated presentations of this stimulus. Nunes et al. [57] analyzed habituation during late childhood and the end of adolescence in male and female SHRs. The authors observed a sex and age difference in habituation, with female SHRs showing lack of habituation from childhood onwards, and male SHRs showing a lack of habituation in adolescence. These difficulties observed in female habituation, however, were overturned by treatment with caffeine (0.3 g/L) during childhood.

##### Working Memory

The object recognition task is recognized as a working-memory task, relies on the animal’s natural tendency for novelty, and tests the ability to discriminate between familiar and unfamiliar objects. França et al. [56] assessed working memory using an adapted version of the object recognition task, conducted in an open field during three different phases: habituation, sample and discrimination. Although the study results indicated that the disruption of the short-term recognition memory persisted into adulthood, the association of caffeine (0.3 mg/mL) and exercise during adulthood and adolescence improved short-term recognition memory in the SHR strain. Nunes et al. [57] also carried out the novel object recognition test and observed similar recognition memory disturbances in adolescent SHRs of both sexes. Nonetheless, caffeine intake (0.3 g/L) restricted to childhood restored recognition memory in adolescent SHRs of both sexes. To evaluate the potential of caffeine in ADHD therapy, Pires et al. [59] treated female WKY rats and SHR with caffeine (3 mg/kg, i.p.) for 14 consecutive days during the prepubertal period. The animals were tested during the object recognition test in the course of adulthood. While WKY rats discriminated between all the used objects, the SHRs were unable to differentiate between pairs of objects with subtle structural differences. Nonetheless, caffeine or MPD chronic treatment improved the deficits in object recognition in SHR. Pires et al. [62] showed, for the first time, the significant impairment of SHRs’ short-term object-recognition ability in comparison with WKY rats. They further investigated the effects of caffeine (1, 3 or 10 mg/kg), 30 min before the sample phase, on the performance of WKY rats and SHR of both sexes in the object recognition task. The injection of caffeine (1, 3 or 10 mg/kg, i.p.) improved the discrimination index of female SHRs, while the highest tested dose of caffeine (10 mg/kg, i.p.) increased the discrimination index of male SHRs.

##### Spatial Learning

The water maze task is a behavioral procedure widely used with rodents to study spatial learning or spatial memory. Prediger et al. [60] used a circular swimming pool to assess the effect of caffeine administration on spatial learning deficit in SHRs. Adult female WKY rats and SHRs were treated with caffeine (1–10 mg/kg i.p.) before or immediately after training, or before the test session. Spatial learning deficit in SHR was improved through the pre-training administration of caffeine (1–10 mg/kg i.p.). SHR test performance was not altered by the post-training administration of caffeine (3 mg/kg i.p.), although WKY rats’ memory retention was increased. Although França et al. [25] observed procedural memory impairment in adolescent SHRs during a cued version of the water maze, these normalized in adulthood.

##### Spatial Short-Term Memory

Given the willingness of rodents to explore new environments, the Y-Maze Test is widely used for testing the conditions affecting memory and learning. Pandolfo et al. [51] assessed SHRs’ spatial short-term memory, using a Y-maze paradigm. When compared with WKY rats, the control group SHRs displayed a spatial learning deficit. Importantly, treatment with caffeine (2 mg/kg, i.p.) during adolescence improved SHR memory impairment. Nunes et al. [57] evaluated spatial memory in male and female SHRs using the Y-maze task at PND 53. Female SHRs showed worsened spatial memory. Although caffeine (0.3 g/L) showed effectiveness against recognition memory deficiency in males and females, only female SHRs increased the number of entries in the novel arm following caffeine treatment, from PND 15 to 55, and showed spatial memory recovery.

#### 3.2.4. Olfactory Discrimination

França et al. [56] assessed the effects of caffeine consumption (0.3 mg/mL) and physical exercise on running on wheels over 6 weeks, during either adolescence (30 days old) or adulthood (4–5 months old), by means of SHR during the olfactory discrimination test. Besides providing the first evidence of deficits in olfactory discrimination in both adolescent and adult SHRs, the authors showed how caffeine, together with physical exercise, was able to restore olfactory discrimination ability in these animals during adolescence or adulthood.

#### 3.2.5. Blood Pressure

França et al. [56] measured systolic blood pressure using the tail-cuff method in a non-invasive manner. For animals treated during adolescence, the systolic arterial pressure was measured before (basal values) and 14, 28, and 42 days after beginning the treatment, before the behavioral tests. For the rats subjected to caffeine treatment and physical exercise during adult life, two measurements were taken, one before the protocols (basal values) and the other after the last behavioral task. Notably, the hypertensive phenotype was not significantly altered by caffeine (0.3 mg/mL) or exercise. When applied from adolescence, caffeine and exercise had no effect on the development of hypertension and at 42 days of treatment (72 days of age), all the SHRs were hypertensive. For adult animals that were already hypertensive at the beginning of the treatment, no further significant differences between groups were observed. To investigate whether the SHRs’ cognitive deficits could be directly associated with hypertension, Pires et al. [59] measured the effects of chronic caffeine administration (3 mg/kg, i.p.) during the prepubertal period on the arterial blood pressure of adult female WKY rats and female SHRs. The SHRs were hypertensive in comparison to the WKY control rats. The chronic administration of caffeine during the prepubertal period, at the same doses that reversed the cognitive deficits of adult SHR (3 mg/kg, i.p.), did not cause significant changes in blood pressure values in adulthood SHR and WKY rats. Again, Pires et al. [62] measured blood pressure after caffeine treatment to investigate whether the cognitive deficits of SHR could be directly related with hypertension. Accordingly, the arterial blood pressure (mmHg) of female WKY rats and female SHRs were measured 30 min after treatment with caffeine (1, 3, or 10 mg/kg, i.p.). As expected, the SHRs were hypertensive in comparison with the WKY control rats. However, the administration of the same doses of caffeine, which was able to improve the object discrimination deficits of the SHRs, did not significantly alter the mean arterial pressure of either the WKY rats or the SHRs. In a similar vein, Prediger et al. [60] measured the arterial blood pressure (mm Hg) of adult female WKY rats and SHRs 30 min after the injection of caffeine (1, 3 or 10 mg/kg, i.p.). Although the SHRs presented a significantly higher mean arterial pressure compared to the WKY control rats, treatment with caffeine did not significantly alter the mean arterial pressure of either the WKY or SHR groups. Caffeine was consequently able to improve the spatial learning deficits of the SHRs without varying their hypertensive state, showing that cognitive impairment in SHR might not be entirely explained by hypertension.

#### 3.2.6. Body Weight

Pires et al. [59] measured the effects of chronic caffeine treatment during the prepubertal period on the body weight of juvenile and adult female WKY rats and female SHR. The body weights of the WKY rats and SHRs was were accordingly measured every 2 days during the treatment (14 days) with caffeine (1, 3, or 10 mg/kg, i.p.). The body weight of the adult rats also recorded during the performance of the object recognition task. Statistical comparisons indicated that juvenile rats from the SHR strain presented significantly lower mean body weight than the juvenile WKY rats. Notably, chronic treatment with caffeine did not alter the body weight of the evaluated rat strains. During adulthood, similar results for the body weight of the animals were found. Although significant strain differences were observed, chronic treatment with caffeine throughout the prepubertal period did not alter the final body weight of the animals in adulthood (regardless of strain). Likewise, Pandolfo et al. (2013) [51] found no weight differences among groups following caffeine treatment (2 mg/kg, i.p.).

#### 3.2.7. Neurobiology

##### Brain Levels of Synaptosomal-Associated Protein-25

França et al. [56] evaluated the effects of caffeine consumption (0.3 mg/mL in drinking water) and physical exercise on running wheels by measuring the brain levels of monoamine, using high-performance liquid chromatography, for 6 weeks. Regarding prefrontal cortex SNAP-25 levels, a statistical analysis revealed a significant increase in SNAP-25 levels in the prefrontal cortex in the group submitted to the combination of caffeine consumption with physical exercise. Regarding hippocampus SNAP-25 levels, the statistical analysis indicated a significant increase in hippocampal SNAP-25 levels selectively in animals submitted to the combination of caffeine consumption with physical exercise.

##### Brain Levels of Syntaxin

SNAP-25 is a component of the soluble N-ethylmaleimidesensitive factor attachment protein receptor (SNARE) complex, which is critical in regulating synaptic vesicle fusion and neurotransmitter release, along with syntaxin 1. Regarding prefrontal cortex syntaxin levels, a statistical analysis performed by França et al. (2020) [56] revealed a significant increase in syntaxin levels in the prefrontal cortex selectively in the group submitted to the combination of caffeine consumption and physical exercise. Regarding hippocampus syntaxin levels, the statistical analysis indicated a main effect of treatment with a marginal effect for treatment versus exercise interaction.

##### Brain Levels of Serotonin

França et al. [56] measured the effects of caffeine consumption and physical exercise throughout adolescence on serotonin (5-hydroxytryptamine, 5-HT) through high-performance liquid chromatography (HPLC). A statistical analysis performed by the authors showed that the combination of caffeine consumption and physical exercise during adolescence increased 5-HT levels in the prefrontal cortex of SHRs. Concerning hippocampal 5-HT levels, statistical comparisons showed that caffeine consumption and physical exercise, alone or in combination, significantly augmented hippocampal 5-HT levels.

##### Brain Levels of Dopamine

França et al. [56] evaluated dopamine levels in the prefrontal cortex, hippocampus, and striatum by using HPLC. Dopamine levels were not detectable in the hippocampus. Although a significant effect of treatment was observed in the prefrontal cortex, no significant effects were observed for exercise or their interaction. Statistical comparisons indicated no significant differences between groups in the levels of dopamine in the prefrontal cortex. Notably, the statistical analysis revealed significant effects of treatment, exercise, and their interaction on striatal dopamine levels. Subsequent statistical comparisons showed that caffeine intake and physical exercise, alone or in combination, significantly augmented striatal dopamine levels.

##### Dopamine Transporter Density

Pandolfo et al. [51] examined if the cognitive and attentional deficits of SHR and their attenuation by caffeine treatment were associated with alterations in the density of DAT in frontocortical and striatal terminals. The number of animals analyzed was four in the WKY control group, four in the WKY caffeine-treated group, three in the SHR control group, and four in the SHR caffeine-treated group. Statistical analysis showed a significant effect of the interaction between strain and treatment in the density of DAT in striatal and frontocortical synaptosomes. Consequently, DAT density was increased in both SHR brain areas of SHR and, significantly, caffeine treatment (2 mg/kg) during adolescence attenuated this enhanced DAT density in both brain areas of the SHRs, while caffeine treatment had no effect on the WKY rats.

##### Dopamine Uptake

Pandolfo et al. [51] tested whether a higher frontocortical density of DAT in SHR was complemented by an augmented uptake of dopamine. The authors directly measured dopamine uptake by synaptosomes. The number of animals was four per group. Both frontocortical and striatal synaptosomes from the SHRs took up almost the double amount of (^3^H) dopamine during the 3 min incubation period than the synaptosomes from the WYK rats. Remarkably, chronic treatment with caffeine (2 mg/kg, i.p.) significantly reduced the dopamine uptake by synaptosomes from both brain areas in the SHRs when compared to vehicle-treated SHRs, while caffeine had no effect on the WKY rats.

##### AdenosineA_2A_ Receptor Density

The effects of chronic caffeine intake are generally attributed to the antagonism of A_2AR_. Consequently, Pandolfo et al. [51] compared the density of A_2AR_ in striatal and frontocortical terminals from SHR or WKY rats treated with caffeine or saline. The number of animals analyzed was four in the WKY control group, three in the WKY caffeine-treated group, four in the SHR control group, and four in the SHR caffeine-treated group. Statistical analysis indicated a significant effect of the interaction between strain and treatment on A_2AR_ density both in the striatum and in the frontal cortex. Notably, fronto-cortical nerve terminals in the SHRs displayed more colocalization between A_2AR_ and synaptophysin immunoreactivities than in the WKY rats. This provided the first direct demonstration of the presence of A_2AR_ in fronto-cortical nerve terminals, and the first indication that A_2AR_ density is improved in SHRs.

##### Colocalization of Dopamine Transporter and Adenosine A_2A_ Receptors

Chronic treatment with caffeine is proposed to operate through A_2AR_ and was shown to affect DAT density and function. Pandolfo et al. [51] proved the colocalization of A_2AR_ and DAT in striatal and frontocortical nerve terminals. The number of animals analyzed was three in the WKY control group, four in the WKY caffeine-treated group, three in the SHR control group and three in the SHR caffeine-treated group. In the striatum, statistical analysis revealed a significant effect of strain on the colocalization of A_2AR_ and DAT immunoreactivities, and a subsequent comparison exhibited that nerve terminals from vehicle-treated SHR displayed a significantly lower colocalization of A_2AR_ and DAT in comparison with vehicle-treated WKY. In the frontal cortex, a statistical analysis revealed no significant effect of strain or treatment on the colocalization between A_2AR_ and DAT.

##### Brain-Derived Neurotrophic Factor

Nunes et al. [57] examined the effects of caffeine (0.3 g/L) administered from childhood onwards in the BDNF and its related proteins in both sexes of SHR rats. BDNF and its related proteins were therefore evaluated in the hippocampus of WKYs and SHRs of both sexes at PND 55. A statistical analysis revealed a significant effect of strain on BDNF levels, while the precursor form (proBDNF) remained unaltered. The TrkB receptor full length (TrkB-FL), phospho-TrkB, and truncated-form TrkB receptors were immunodetected in the hippocampuses of the WKYs and SHRs of both sexes. A statistical analysis revealed a significant effect of strain on the truncated form and also on phospho-TrkB. Furthermore, the transcription factor CREB was not altered either by strain or sex, although its phosphorylated form (phospho-CREB) was increased in the SHR hippocampus from both sexes. Finally, Nunes et al. [57] evaluated the impact of caffeine only on the BDNF levels and TrkB receptors (TrkB-FL, phospho-TrkB, and TrkB-T). Caffeine administered from PND 15 up to PND 55 (caff/caff) reduced the BDNF levels in the hippocampuses of SHR male rats, whereas the BDNF levels were unaltered in the SHR female rats in both schedules of treatment. In the male rats, caffeine in both schedules of treatment did not change either TrkB-FL or TrkB-T levels, whereas female SHRs showed reduced TrkB-FL and TrkB-T forms as a consequence of caffeine treatment. Neither the increased phospho-TrkB nor the CREB were modified in the hippocampuses of the SHRs following caffeine treatment.

##### Neuronal Development In Vitro

Alves et al. [63] investigated caffeine’s in vitro effects at the neuronal level. At first, SHR and WKY rats’ cultured frontal cortical neurons were immunostained for MAP-2 during in vitro development. Later on, somatodendritic analyses were performed, measuring branch point number, root number, and maximal and total neurite length. Neurons from the SHRs displayed fewer differentiation patterns, including neurite branching, shorter maximal neurite length, and decreased axonal outgrowth. Following a 24 h period of caffeine incubation (30 μM), the SHR neurons showed an inferior percentage of zero branch points, and a superior percentage of two branch points. A trend toward a superior percentage of one-branch-point-neurons was observed for SHR neurons following treatment with caffeine. Caffeine also promoted a rise in the total and maximal neurite length in neurons from both strains. PKA or PI3K inhibitor were subsequently used to study whether one of the transducing systems activated by adenosine receptors, and in the neuronal differentiation, are responsible for the effects produced by caffeine. PKA inhibitor KT5720 (5 μM) did not change caffeine’s ability to augment the percentage of SHR neurons with more branch points. Caffeine’s effect on the recovery of the total neurite length of the SHR neurons was obstructed by PKA inhibitor. Comparable results were seen for maximal neurite length, in which PKA inhibitor completely decreased caffeine’s effects. Finally, LY294002 (50 μM) was used as an inhibitor of PI3K and its presence blocked caffeine’s effect on the increase in the number of branch points in SHR neurons. Furthermore, caffeine’s effect on the prevention of reductions in the total neurite length were eliminated in the presence of PI3K inhibitor. Similar results were found for the maximal neurite length. The number of roots was also reduced by PI3K inhibitor in SHR neurons.

## 4. Discussion

ADHD is characterized by symptoms including attention deficits, impulsivity, and hyperactivity [3,4] that frequently persist throughout life [1,2,6]. Prefrontal cortex function modulation and attentional/behavioral regulation depends on the optimal release of signalling molecules such as NE, DA [24,25], as well as 5-HT, GLU, or ACh [26,27,28]. In this respect, genes, including the DAT or the DRD4 [31,32] or the SERT, the SNAP-25, and the BDNF [29,30], might play a role in causing ADHD. Therefore, agents that can lead to the optimal balance of these organic compounds are hypothetically beneficial in patients with ADHD by mainly returning prefrontal activity to adequate functional levels [18,33]. In this sense, it has long been discussed whether caffeine could become an effective pharmacological compound for the management of symptoms of ADHD [64,65].

This systematic review analyzed 13 animal studies that investigated the effects of caffeine on the modulation of ADHD-like symptoms. Overall, the reviewed results show that caffeine treatment increases attention and improves learning, memory, and olfactory discrimination without altering blood pressure and body weight.

Regarding attention, caffeine treatment improved the attentional and behavioral flexibility of SHRs [51], the spatial attention of 6-OHDA lesioned rats [53], and SI in ICR mice [52] during adolescence. Caffeine treatment improved the reaction time of LE and CD rats [55] and focus and attention in zebrafish [54] during adulthood.

Regarding learning and memory, caffeine treatment plus physical exercise during adulthood and adolescence improved working memory in SHRs [56]. In the same vein, caffeine treatment alone restored non-associative learning in female SHRs [57], improved working memory in SHRs [59], female SHRs [62], and adolescent SHRs [57]. The administration of caffeine improved spatial learning deficit in SHRs, increased memory retention in WKY rats [60], and improved spatial short-term memory in SHRs [51] and female SHRs [57].

Concerning olfactory discrimination, caffeine treatment, together with physical exercise, was able to restore olfactory discrimination in SHRs during adolescence or adulthood [56]. Concerning blood pressure, caffeine treatment did not alter the hypertensive phenotype in SHR [60,62] during adolescence or adult life [56], nor during the adult female SHR prepubertal period [59]. Finally, caffeine treatment did not alter body weight in SHRs [51,59].

If we are ever to acquire a truly in-depth understanding of ADHD pharmacotherapy, we need to face the following question: Does caffeine deserve a place in the battery of pharmacological agents for ADHD treatment, particularly during adolescence? Although previous meta-analyses [64] and reviews [65] were unable to provide any recommendations for adolescents diagnosed with ADHD, due to a lack of data, our reviewed results provide updated preclinical evidence and support the therapeutic potential of caffeine to improve attention, learning, memory, or olfactory discrimination in ADHD, especially during adolescence.

Beyond its clear effects on improving performance in tasks requiring attention, learning, memory and olfactory discrimination, without altering blood pressure and body weight, the implication of caffeine in modulating ADHD-like hyperactivity symptoms remains controversial. Indeed, caffeine treatment plus physical exercise did not affect locomotor activity in SHRs [56]. In a similar manner, caffeine treatment alone did not alter locomotion in SHR [51,59,60], preadolescent SHR [57], or young LDLr mice [58]. Nonetheless, caffeine treatment did increase locomotor activity in adolescent female SHRs [57], zebrafish [54]. Furthermore, it produced an increase related to dose in locomotion in CD rats and a significant attenuation of CGS-21680-induced hypolocomotion in CD rats [55], and it attenuated locomotor activity in middle-aged LDLr mice [58] and 6-OHDA lesioned rats throughout the prepubertal period [53]. This apparent discrepancy may have resulted from caffeine ‘s promotion of different effects according to age and sex. In this regard, Nunes et al. [57] suggested that the intake of caffeine from the childhood period onwards may aggravate hyperactivity in females, if the consumption continues up to the adolescence period. Szczepanik et al. [58] linked the age-dependent effect induced by caffeine with the idea that the blockade of adenosine A_1_/A_2A_ receptors attempts to renormalize a potentially maladaptive system [66], with age an important escalating factor in mice. In a different study, Ruiz-Oliveira et al. [54] proposed that caffeine-induced bursts of locomotion may be caused by a decrease in fatigue [67] rather than by an anxiogenic response. Importantly, the attenuation of motor activity by caffeine consumption was determined as a natural effect of growth rather than an effect of caffeine intake by Caballero et al. [53].

In terms of impulsivity, although acute pretreatment with caffeine increased the number of large-reward choices made by SHRs, chronic treatment with caffeine increased the impulsive phenotype and decreased choices of large rewards by SHRs [61]. This discrepancy may be explained by previous studies performed on animal models of brain diseases, showing that while acute treatment acts mainly on A_1_ receptors, chronic treatment acts mainly on A_2A_ receptors [68]. Leffa et al. [61], in this direction, underscored the ability of the adenosine modulation system to control behavioral inhibition.

Besides reviewing animal studies deciphering the effects of caffeine in the modulation of ADHD-like symptoms, we reviewed for the first time animal studies examining the effects of caffeine and adenosine receptors on neurons isolated from SHRs, at the neuronal level.

In this respect, treatment with caffeine and physical exercise during the adolescence period augmented the quantity of SNAP-25, syntaxin, and serotonin in the prefrontal cortex and the hippocampus, as well as striatal dopamine quantity, in SHRs [56]. In a similar manner, caffeine treatment alone during the adolescence period attenuated the improvement in DAT density in the fronto-cortical and striatal terminals of SHRs and diminished the dopamine uptake by synaptosomes from SHRs’ fronto-cortical and striatal terminals [51]. Furthermore, Pandolfo et al. [51] demonstrated that fronto-cortical nerve terminals are provided with AdenosineA_2A_ receptor, the target of chronic caffeine exposure, whose density was found to be increased in SHRs. Caffeine treatment normalized BDNF levels in the hippocampuses of SHR males, while the same treatment normalized TrkB receptors TrkB-FL and TrkB-T SHR in the hippocampuses of SHR females [57]. Finally, neurons from SHRs showed an inferior number of zero-branch points, and a superior number of two-branch-points-neurons following in vitro caffeine treatment consisting of 24 h of caffeine incubation. After treatment with caffeine, an increase in the total and maximal neurite length and a tendency toward a superior number of one-branch-point neurons was also observed for SHR neurons. The effect of caffeine on increasing maximal neurite length, and on recuperating the entire neurite length of neurons from SHR, was entirely blocked by PKA inhibitor. LY294002, as an inhibitor of PI3K, blocked caffeine’s effects on the increase in the amount of branch points in SHR neurons. Finally, the effect of caffeine on the prevention of reductions in the total neurite length, increasing maximal neurite length, and the number of roots was eradicated by the presence of PI3K inhibitor in SHR neurons [63].

## 5. Conclusions

Overall, our reviewed data suggest that caffeine is a possible adjuvant pharmacological strategy for the treatment of ADHD. The compiled preclinical data support the notion that caffeine improves ADHD-like symptoms of inattention and its related learning and memory impairments without affecting blood pressure and body weight. Our results are supported at the neuronal/molecular level, and strengthen the hypothesis that the cognitive effects of caffeine found in animal models of ADHD could be translated to humans diagnosed with the disorder, particularly during adolescence. Nonetheless, caution is needed when extrapolating potential effects identified in animal studies to human patients. In this work, studies that explored caffeine’s effects on locomotor activity and impulsivity were contradictory, raising discrepancies that require further clarification. Although we consider that the reviewed results in this manuscript can potentially impact the scientific, pre-clinical, and clinical community and expand our knowledge regarding ADHD, more studies should be performed to validate our present knowledge while offering prospective clues to support caffeine as a therapeutic approach for the treatment of ADHD.

## Figures and Tables

**Table 1:** Summary of included studies.

Author/s & Year	Species, Strain, Sex & Sample (*n*)	Animal Model	Age	Independent Variables	Caffeine Treatment	Behavioral Tests/ Type of Stress	Dependent Variables	Main Results
Szczepanik et al., 2016	Mice C57Bl/6 wild-type (8) LDLr (8) Female	Genetic (LDLr)	3 months 8 months	Treatment (caffeine or vehicle) Strain (C57Bl/6 wild type or LDLr)	10 mg/kg oral route Chronic treatment (21 days)	Open-field arena	Spontaneous locomotor activity (total distance travelled) Anxiety (time in the center) Exploratory behavior (visual inspection of the occupation plot)	- LDLr mice travelled greater distances than the C57BI/6 wild type mice during the 5 min period of analysis. - Caffeine treatment induced a renormalization effect in 8 month-old mouse locomotion. - Caffeine treatment was unable to modify the hyperlocomotion observed in 3 month-old LDLr mice. - All animal groups spent a similar amount of time in the center of an open field. - Similar exploratory behavior between groups.
Higgins et al., 2007	Rats LE (15–16) CD (12–16) Male	Not used	Not specified	Treatment (caffeine, SCH412348, KW-6002, DPCPX, CGS-21680, amphetamine) Strain (LE or CD)	1 mL/kg i.p. route One dose, prior testing	Five-choice serial reaction time task Locomotor activity test	Selective attention (Correct/incorrect trials, omissions, premature and perseverative responses, choice accuracy, correct/incorrect, and magazine latency) Hypolocomotion (distance travelled)	- Caffeine, SCH 412348 and KW-6002 augmented time reaction in LE and CD, without effect on accuracy. - Effects of SCH 412348 were at doses that were not overtly psychostimulatory. - CGS-21680 reduced speed reaction and augmented omissions. A CGS-21680 lower dose reduced the increased premature response caused by amphetamine. - Caffeine’s attentional-enhancing effects were facilitated through A_2A_ receptor blockade. Selective A_2A_ receptor antagonists could be included as a potential therapy for ADHD.
Ruiz-Oliveira et al., 2019	Zebrafish wild-type (40) Male Female	Not used	4 months	Treatment (caffeine or vehicle)	10 mg/L 50 mg/L drinking water Chronic treatment (14 days)	Discrimination task	Conditioned learning ability (average swimming speed, intergroup freezing, maximum speed, time spent in each area, latency to enter each area)	- 0 and 10 mg/L caffeine groups spent most of the time close to the target. - 10 mg caffeine group had the shortest latency to reach the target. - 0 and 10 mg/L caffeine groups increased the average speed and distance travelled. - Caffeine exposure at low doses seems to enhance visual cue discrimination and zebrafish performance.
Prediger et al., 2005	Rats WKY (7–8) SHR (7–8) Female	Genetic (SHR)	3 months	Treatment (caffeine or vehicle) Strain (WKY or SHR)	1.3 mg/kg 10 mg/kg i.p. route One dose, prior testing	Water maze task	Spatial learning (escape latency, distance travelled, swimming speed) Mean arterial pressure	- SHR needed a larger amount of trials during the training session to learn the spatial information, although a similar profile to that of WKY rats during the test session, showing a selective spatial learning deficit. - Caffeine’s pre-training administration enhanced SHRs’ spatial learning deficit. - Caffeine’s post-training administration did not enhance SHRs’ test performance, although it improved WKY rats’ memory retention. - Mean blood pressure was not altered by caffeine.
Pires et al., 2009	Rats WKY (15) SHR (18) Male Female	Genetic (SHR)	3 months	Treatment (MPD, DPCPX, caffeine, ZM241385 or vehicle) Strain (WKY or SHR)	1 mg/kg 3 mg/kg 10 mg/kg i.p. route One dose, prior testing	Object recognition task	Object recognition (investigation time, discrimination time) Mean arterial pressure	- SHR only discriminated between the most structurally distinct pairs of objects. - Pre-training administration of MPD, caffeine, the selective adenosine receptor antagonists DPCPX and ZM241385, or the association of ineffective doses of DPCPX and ZM241385, improved the performance of SHR in the object-recognition task. - The administration of the same doses of MPD and caffeine did not significantly alter the mean arterial pressure of either WKYs or SHRs.
Pires et al., 2010	Rats WKY (37) SHR (38) Female	Genetic (SHR)	25/38 days	Treatment (caffeine, MPD or vehicle) Strain (WKY or SHR)	3 mg/kg i.p. Route. Chronic treatment (14 days)	Object recognition task	Object recognition (investigation time, discrimination time) Spontaneous locomotor activity Mean arterial pressure Body weight	- WKY rats distinguished all the items. SHRs were unable to distinguish pairs of items with slight structural alterations. - Caffeine or MPD chronic treatment enhanced SHR item-recognition deficits. The same treatments impaired the adult WKY rats’ short-term object recognition ability. - Effects were independent of variations in locomotion, arterial blood pressure, and body weight.
Caballero et al., 2011	Rats 6-OHDA lesioned (9) Saline- lesioned (9) Male Female	Physical trauma (6-OHDA lesioned)	25 days	Treatment (caffeine or vehicle)	1 mg/mL drinking water Chronic treatment (14 days)	Olton maze behavioral assay	Motor behavior (number of arms crossed) Attention behavior (total number of arms walked, and total number of arms walked until one was repeated)	- Caffeine treatment significantly improved 6-OHDA lesioned rats’ attention deficit. - After caffeine consumption, no changes were found in motor activity.
Pandolfo et al., 2013	Rats WKY (16) SHR (16) Male	Genetic (SHR)	24 days	Treatment (caffeine or vehicle (saline)) Strain (WKY or SHR)	2 mg/kg i.p. route Chronic treatment (twice daily for 21 days)	Attentional-set shifting; anxiety-related behavior; Y maze; locomotion -related behavior	Attention (regressive and never- reinforced errors, perseverative errors, total number of trials required before reaching 10 correct consecutive choices) Locomotion and anxiety (number of peripheral squares crossed, number of central squares crossed, percentage of central locomotion) Spatial recognition (number of entries; time spent per arm; random exploration)	- SHRs were hyperactive and showed poorer performance in the attentional set-shifting and Y-maze paradigms, displayed increased dopamine transporter density, and increased dopamine uptake in frontocortical and striatal terminals. - Chronic caffeine treatment improved memory and attention deficits, and normalized dopaminergic function in SHR. - First indication of adenosine A_2A_ receptors (A_2A_R) in nerve terminals in frontal cortex. - First evidence that A_2A_R density is improved in SHR.
Ouichi et al., 2013	Mice ICR (9) Male Female	Physical trauma (SI)	4 weeks	Treatment (MPD and caffeine) SI	0.5–1 mg/kg i.p. route One dose, prior testing	Water-finding test; aggression; modified Y-maze test; novel object recognition test; fear-conditioning test	Spatial attention (entering & drinking latency) Aggression (duration of wrestling) Spatial recognition (time spent in the new arm; total time exploring objects) Fear conditioning (freezing behavior)	- SI rats showed deficits in spatial attention on the water-finding test. Re-socialized did not reduce deficit in spatial attention. SI effect on spatial attention revealed no difference in gender or correlation with aggressive behaviour. - SI impaired conditional and contextual fear memory. - MPD and caffeine enhanced deficits in SI-induced latent learning in a manner that was reversible with cholinergic but not dopaminergic antagonists.
Nunes et al., 2018	Rats WKY (5–15) SHR (5–15) Male Female	Genetic (SHR)	15 days 28 days 50 days	Treatment (caffeine/water, caffeine/caffeine or water) Strain (WKY or SHR)	0.3 g/L drinking water Until PND 28	Open-field test; Novel object recognition; Y maze task	Open field test (travel distance periphery) Habituation (total travelled distance in the open field) Spatial recognition- Y maze and object recognition (exploration, discrimination ratio, number of entries, time spent in novel arm, total number of entries in three arms)	- Adolescent SHR from both sexes displayed hyperlocomotion, recognition, and spatial memory disturbances. Females displayed a lack of habituation and deteriorated spatial memory. - Caffeine was effective at improving recognition memory damage in both sexes. - Spatial memory was improved only in female SHRs. - Female SHRs displayed impaired hyperlocomotion following caffeine treatment. - SHRs of both sexes presented increases in BDNF, truncated and phospho-TrkB receptors, and phospho-CREB levels in the hippocampus. - Caffeine normalized BDNF in males and truncated TrkB receptor in both sexes.
Leffa et al., 2019	Rats WKY (7–9) SHR (7–9) Male	Genetic (SHR)	60/65 days 24 days	Treatment (WIN, AM251, caffeine or vehicle) Strain (WKY or SHR)	2 or 5 mg/kg i.p. route Acute pretreatment, one dose Chronic treatment (21 days)	Tolerance to delay of reward; T maze	Impulsive behavior (tolerance to delay of reward)	- WIN treatment decreased large reward choices and AM251 treatment increased large reward choices in SHR. - Acute caffeine pretreatment blocked WIN effects. - Chronic caffeine treatment increased the impulsive phenotype and potentiated the WIN effects. - Cannabinoid and adenosine receptors modulate impulsive behavior in SHR.
Alves et al., 2020	Rats-pregnant SHR (40–70) WKY (40–70) Female	Genetic (SHR)	In vitro	Treatment (caffeine, DMSO, LY294002, adenosine selective agonist and antagonists) Strain (WKY or SHR)	Caffeine incubation (30 µM) One dose	No behavioral task	Morphological alterations (singling, neurite branching)	- SHR neurons displayed less neurite branching, shorter maximal neurite length and decreased axonal outgrowth. - Caffeine recovered neurite branching and elongation from SHR neurons via PKA and PI3K signaling, - A_2A_R agonist (CGS 21680) promoted more neurite branching via PKA signaling. - The selective A_2A_R antagonist (SCH 58261) was efficient at recovering axonal outgrowth from SHR neurons through PI3K and not PKA signaling.
França et al., 2020	Rats WKY (9) SHR (11) Male	Genetic (SHR)	30 days 4–5 months	Treatment (caffeine or water) Physical exercise Strain (WKY or SHR)	0.3 mg/mL, drinking water One dose	Olfactory discrimination; Open field; Object recognition; Water maze	Olfactory discrimination (time spent in compartments, numbers of crossings) Locomotor activity (total distance, time spent in the central zone) Short-term memory (total time spent exploring the objects, discrimination index) Working and procedural memories (escape latency)	- SHR showed olfactory and short-term recognition memory deficiencies from adolescence to adulthood, accompanied by lower prefrontal cortex and hippocampus SNAP-25 levels. - Caffeine and physical exercise during adolescence or adulthood repaired the olfactory discrimination ability and enhanced short-term recognition memory in SHRs. - Caffeine consumption and physical exercise during adolescence augmented hippocampus and prefrontal cortex SNAP-25, syntaxin, and serotonin levels, as well as SHRs’ striatal dopamine levels.

Key for abbreviations used: LDLr: low-density lipoprotein receptor, LE rat: Long–Evans rat, CD rat: Cesarean-derived rat, i.p.: intraperitoneally, WKY rat: wistar Kyoto rat, SHR: spontaneously hypertensive rat, MPD: methylphenidate, 6-OHDA: 6-hydroxy-dopamine, A_2A_R: Adenosine A_2A_ receptors, ICR mice: Institute of Cancer Research mice, SI: social isolation, PND: postnatal day, BDNF: brain-derived neurotrophic factor, SNAP-25: synaptosomal-associated protein 25.

## Data Availability

Not applicable.
